# Suppressive Effect of Insulin on the Gene Expression and Plasma Concentrations of Mediators of Asthmatic Inflammation

**DOI:** 10.1155/2015/202406

**Published:** 2015-01-06

**Authors:** Husam Ghanim, Kelly Green, Sanaa Abuaysheh, Manav Batra, Nitesh D. Kuhadiya, Reema Patel, Antoine Makdissi, Sandeep Dhindsa, Ajay Chaudhuri, Paresh Dandona

**Affiliations:** ^1^Division of Endocrinology, Diabetes and Metabolism, State University of New York at Buffalo and Kaleida Health, 115 Flint Road, Williamsville, NY 14221, USA; ^2^Division of Endocrinology and Metabolism, Texas Tech University Health Sciences Center, 701 W 5th Street, Odessa, TX 79763, USA

## Abstract

*Background and Hypothesis*. Following our recent demonstration that the chronic inflammatory and insulin resistant state of obesity is associated with an increase in the expression of mediators known to contribute to the pathogenesis of asthma and that weight loss after gastric bypass surgery results in the reduction of these genes, we have now hypothesized that insulin suppresses the cellular expression and plasma concentrations of these mediators. *Methods*. The expression of IL-4, LIGHT, LTBR, ADAM-33, and TSLP in MNC and plasma concentrations of LIGHT, TGF-*β*1, MMP-9, MCP-1, TSLP, and NOM in obese patients with T2DM were measured before, during, and after the infusion of a low dose (2 U/h) infusion of insulin for 4 hours. The patients were also infused with dextrose or saline for 4 hours on two separate visits and served as controls. *Results*. Following insulin infusion, the mRNA expression of IL-4, ADAM-33, LIGHT, and LTBR mRNA expression fell significantly (*P* < 0.05 for all). There was also a concomitant reduction in plasma NOM, LIGHT, TGF-*β*1, MCP-1, and MMP-9 concentrations. *Conclusions*. Insulin suppresses the expression of these genes and mediators related to asthma and may, therefore, have a potential role in the treatment of asthma.

## 1. Introduction

We have recently demonstrated that the chronic inflammatory states of obesity and type 2 diabetes are associated with an increase in the cellular expression and the plasma concentrations of mediators involved in the pathogenesis of asthma, consistent with the increase in the prevalence of asthma in these conditions [1]. These data provide the first mechanistic link between obesity and asthma, beyond the obvious effects on lung volumes. Furthermore, our data provided the first evidence that, following gastric bypass surgery and weight loss, there was a significant reduction in these mediators including IL-4, the key TH-2 cytokine associated with asthma [2].

Other inflammatory mediators, which play a role in asthma, include matrix metalloproteinases (MMP), including MMP-9 and ADAM-33 (a disintegrin and metalloproteinase-33); chemokines including eotaxins, RANTES, and MCP-1; chemokine receptors, CCR-3 for eotaxin, CCR-5 for RANTES, and CCR-2 for MCP-1 [3, 4]. More recently, it has been shown that LIGHT and its receptor, lymphotoxin-*β* receptor (LTBR), also participate in asthmatic inflammation by mediating remodeling of bronchi and bronchioles. The remodeling action of LIGHT-LTBR is mediated by TGF*β* which induces fibrosis and also leads to epithelial-mesenchymal transformation [5]. Most recently, thymic stromal lymphopoietin (TSLP) has been shown to contribute to the pathogenesis of asthma and treatment of asthmatic patients with a monoclonal antibody directed against TSLP improves respiratory function [6]. In addition to the possible contribution of these proinflammatory mediators, there is also an increase in nitric oxide (NO) and isoprostane content in exhaled air in asthmatics when compared to normal subjects [7]. The increase in NO is likely due to the activation of iNOS in bronchial macrophages and is thought to relate to clinical activity of asthma [8]. Increased isoprostane generation is a reflection of increased oxidative stress which also characterizes asthmatic inflammation [9]. We have recently shown that obesity and diabetes are associated with an increase in the expression of IL-4, LIGHT, CCR-2, and MMP-9 in MNC and that, following gastric bypass surgery, there is a reduction in the expression of these genes [1]. In addition, there is also a reduction in the expression of ADAM-33 in parallel with weight loss and an increase in insulin sensitivity.

Our previous work has shown that insulin exerts a potent and rapid anti-inflammatory effect inducing a suppression of major transcription factors like NF*κ*B and Egr-1 along with a reduction in the concentration of several proinflammatory mediators in plasma including ICAM-1, MCP-1, MMP-9, PAI-1, and VEGF and the expression of inflammatory cytokines and chemokines in the MNC [10–12]. More recently, a low dose insulin infusion has also been shown to suppress the expression of several toll-like receptors including TLRs 1, 2, 4, 7, and 9; PU.1 DNA binding; and TLR2 protein in MNC from obese patients with T2DM [13]. PU.1 is the major transcription factor modulating the transcription of TLRs. The suppression of TLRs is important in the context of asthma since TLRs are pathogen recognition receptors and bacterial and viral infections are important triggers in the exacerbation of inflammation in asthma [14].

On the basis of the above, we hypothesized that the expression of IL-4, ADAM-33, MMP-9, LIGHT, LTBR, and TSLP and the plasma concentration of LIGHT, NOM, MCP-1, MMP-9, TGF-*β*, and TSLP are suppressed by an intravenous infusion of insulin. These factors/genes have mostly been associated with asthma during the past decade and, therefore, were specifically included in our hypothesis. In addition, these are the genes we investigated in our previous paper based on data from morbidly obese patients before and after gastric bypass surgery [1].

## 2. Methods

### 2.1. Subjects

#### 2.1.1. Insulin Infusion Study

Ten obese patients with type 2 diabetes (T2DM) (5 females and 5 males, age: 47.9 ± 8.9 years; BMI: 39.2 ± 6.5 kg/m^2^) were recruited for a crossover insulin infusion study. The diabetics had a mean HbA1c of 7.0 ± 0.81%. They were on stable oral antidiabetic medications. All patients were on metformin (1-2 g/day) and 6 patients were on sulfonylureas (glyburide or glipizide 5–10 mg/day). After an overnight fast, subjects were infused with insulin (2 U/h) with 5% glucose (100 mL/h) and 20 mEq of potassium chloride per hour for 4 h followed by 2 h of observation. Blood glucose levels were measured every 15 minutes and were maintained at a target level of 80–130 mg/dL. On a separate visit, all subjects were also infused with 5% glucose alone at a rate of 100 mL/h to control for the dextrose infused with insulin. On the third visit, they were infused with normal saline alone at a rate of 100 mL/h for 4 h to control for the volume infused during the insulin or glucose infusions. Since four patients declined participation in the normal saline arm of the study, only 6 patients (4 females and 2 males, age: 41.5 ± 8.2 years, BMI 36.9 ± 6.7 kg/m^2^, and HbA1c of 7.5 ± 1.1%) were infused with saline. Blood samples were collected at baseline and at 2 h, 4 h, and 6 h following the infusion. The protocol was approved by the Human Research Committee of the State University of New York at Buffalo. An informed consent was signed by all subjects.

### 2.2. MNC Isolation

Blood samples were collected in Na-EDTA and carefully layered on Lympholyte medium (Cedarlane Laboratories, Hornby, ON). Samples were centrifuged and two bands separated out at the top of the RBC pellet. The MNC band was harvested and washed twice with Hank's balanced salt solution (HBSS). This method provides yields greater than 95% MNC preparation.

### 2.3. ROS Generation Measurement by Chemiluminescence

Five hundred *μ*L of MNC (2 × 10^5^ cells) was delivered into a cuvette. Luminol was then added, followed by 1.0 *μ*L of 10 mM formylmethionyl leucyl phenylalanine (fMLP) and chemiluminescence was recorded for 5 min using Chronolog Lumi-Aggregometer as previously described [15].

### 2.4. Quantification of mRNA in MNC by RT-PCR

Total RNA was isolated using commercially available RNAqueous-4PCR Kit (Ambion, Austin, TX). RT-PCR was performed using Cepheid Smart Cycler (Sunnyvale, CA), Sybergreen Master mix (Qiagen, CA), and gene specific primers for IL-4, LIGHT, LTBR, TSLP, and ADAM-33 (Life Technologies, MD). All samples were assayed for a group of 4 housekeeping genes: *β*-actin, ubiquitin C, cyclophilin A, and RPS3 (Biosearch Technologies, Inc., Petaluma, CA). A normalization factor based on the values of all housekeeping genes for each sample was calculated by GeNorm software (Qbase) and was used to normalize the expression of the genes of interest.

### 2.5. Plasma Measurements

Glucose concentrations were measured in plasma by YSI 2300 STAT Plus glucose analyzer (Yellow Springs, OH). ELISA was used to measure insulin (Diagnostic Systems Laboratories Inc., Webster, TX), MCP-1, MMP-9, TGF-*β*1, LIGHT (R&D Systems, Minneapolis, MN), and TSLP (Biolegend, San Diego, CA). Plasma concentrations of nitric oxide metabolites (NOM: NO_2_/NO_3_) were measured by Griess reaction using a colorimetric assay kit from R&D Systems (Minneapolis, MN).

### 2.6. Statistical Analysis

Statistical analysis was conducted using SigmaStat software (SPSS Inc., Chicago, IL). All data are represented as mean ± SE. Statistical analysis from baselines was carried out using Holm-Sidak one-way repeated measures analysis of variance (RMANOVA). Dunnett's two-factor RMANOVA method was used for multiple comparisons between different groups. Demographic variables and baseline levels of inflammatory mediators in lean, obese, and obese T2DM patients were compared using Student's *t*-test. *P* < 0.05 was considered significant.

## 3. Results

### 3.1. Insulin and Glucose Concentrations following Insulin Infusion

Mean blood glucose concentrations did not change significantly during the first 4 hours of the insulin, dextrose, or saline infusions but did fall significantly at 6 hr (2 hours following cessation of the infusions) in all groups (Table 1). Blood glucose at baseline and at 4 h was not significantly different between the three groups. Plasma insulin concentration increased from 20.9 ± 10.9 *μ*U/mL to 50.5 ± 22.4 *μ*U/mL (*P* < 0.001) during the insulin infusion while it fell slightly in the dextrose groups from 27.6 ± 5.6 *μ*U/mL to 22.9 ± 6.5 *μ*U/mL at 4 h (NS) and in the normal saline group from 20.6 ± 5.5 *μ*U/mL to 17.9 ± 4.7 *μ*U/mL at 4 h (NS) (Table 1).

### 3.2. Effect of Insulin Infusion on Expression of IL-4, ADAM-33, LIGHT, LTBR, MMP-9, and TSLP

**Figure 1:**
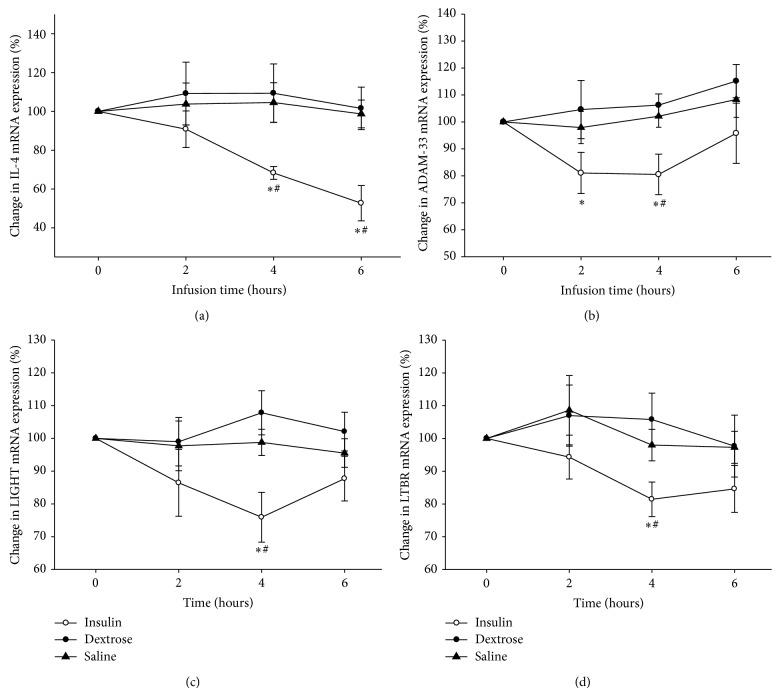
Percent change in (a) IL-4 (b) ADAM-33 (c) LIGHT, and (d) LTBR mRNA expression in MNC following 2 U/hr insulin/dextrose (insulin), dextrose alone (dextrose), or saline alone (saline) infusions for 4 hr in obese T2DM patients. Data is presented as mean ± SE. ^*^ *P* < 0.05, when compared to baseline by one-way RMANOVA; ^#^ *P* < 0.05 compared to control groups by 2-way RMANOVA.

The infusion of insulin led to the suppression of IL-4 mRNA expression in MNC starting at 2 h, increasing at 4 h, and maximizing at 6 h by 44 ± 7% (*P* < 0.05) below the baseline, in spite of the cessation of the infusion at 4 h. (Figure 1(a)). ADAM-33 mRNA expression was suppressed significantly by 20 ± 8% following insulin infusion (*P* < 0.05) (Figure 1(b)). Additionally, insulin infusion suppressed the expression of LIGHT and LTBR expression by 22 ± 9% and 19 ± 7% at 4 hr (*P* < 0.05) (Figures 1(c)-1(d)). There was no significant change in the mRNA expression of MCP-1, TSLP, and MMP-9 in MNC following insulin. In the control arms, infused with dextrose alone or saline, there was no alteration in the expression of IL-4, ADAM-33, LIGHT, and LTBR in MNC.

### 3.3. Effect of Insulin Infusion on Plasma Levels of LIGHT, TGF*β*1, NOM, MCP-1, TSLP, and MMP-9

**Figure 2:**
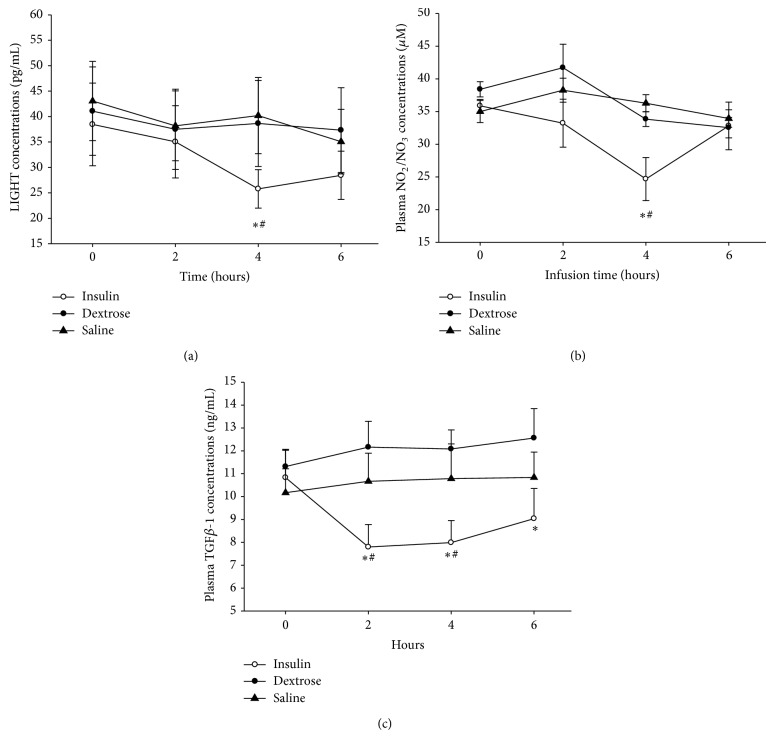
Change in (a) LIGHT, (b) NOM, and (c) TGF-*β*1 concentrations following 2 U/hr insulin/dextrose (insulin), dextrose alone (dextrose), or saline alone (saline) infusions for 4 hr in obese T2DM patients. Data is presented as mean ± SE. ^*^ *P* < 0.05, when compared to baseline by one-way RMANOVA; ^#^ *P* < 0.05 compared to control groups by 2-way RMANOVA.

The infusion of insulin in T2DM patients caused a significant suppression of LIGHT concentrations by 25 ± 5% (from 39.4 ± 10.6 to 27.5 ± 6.4 pg/mL, *P* < 0.05, Figure 2(a)). Following insulin infusion, plasma concentration of NOM (NO_2_/NO_3_) fell by 34 ± 15% at 4 h (*P* < 0.05) (Figure 2(b)). Additionally the infusion of insulin caused a significant suppression of TGF-*β*1 concentrations by 28 ± 6% (from 10.8 ± 1.2 to 7.7 ± 1.0 ng/mL, *P* < 0.05, Figure 2(c)). This suppression occurred in parallel with significant (*P* < 0.05, for all) and consistent reductions in ROS generation by MNC (by 18 ± 5%) and serum levels of MCP-1 (by 15 ± 4%) and MMP-9 (by 14 ± 5%) at 4 h following insulin infusion (Table 1). Plasma TSLP concentrations did not alter after insulin (from 10.0 ± 1.2 to 10.5 ± 1.2 pg/mL at 4 hr). There was no significant change in NOM, LIGHT concentration, or other inflammatory indices following dextrose or saline infusions.

## 4. Discussion

Our data show for the first time that intravenous infusion of insulin leads to the suppression of IL-4 expression in MNC at 2 h by 25% and by 34% at 4 h. A further increase in the suppression of IL-4 by 43% was evident at 6 h in spite of the cessation of the infusion at 4 h. Thus the IL-4 suppressive action of insulin is rapid, potent, and prolonged. This is of interest since a preparation of a monoclonal antibody against the alpha subunit of IL-4 has recently been shown to suppress clinical activity of asthma as reflected in exacerbation rates, FeV_1_, and mean nocturnal awakening rates [16]. In addition, IL-4 antibody reduced exhaled NO and plasma eotaxin concentrations. Our current data show that insulin also reduced plasma concentrations of NO metabolites. The fall of NOM in plasma parallels the reduction of NO in exhaled air as discussed below. We have previously shown that insulin suppresses eotaxin concentrations along with other chemokines and chemokine receptors [12], as also discussed below.

Our current data also show that there was a concomitant suppression of ADAM-33, LIGHT, and LTBR, three other mediators known to be associated with the pathogenesis of asthma. These actions are consistent with our previous observations on the anti-inflammatory effects of insulin as reflected in effects like the suppression of intranuclear NF*κ*B binding, ROS generation, p47^phox^ expression, Egr-1 binding and expression, and plasma ICAM-1, MCP-1, MMP-9, VEGF, and PAI-1 concentrations [10, 11]. Insulin also induces an increase in I*κ*B*α* expression, consistent with its ability to suppress NF*κ*B binding. There was no change in the expression of TSLP.

The infusion of insulin led to the concomitant suppression of the expression of ADAM-33 and plasma concentrations of MMP-9, both of which are matrix metalloproteinases which participate in the pathogenesis of asthmatic inflammation and bronchial remodeling. LIGHT and its receptor LTBR have very recently been shown to be involved in bronchial remodeling and sensitization to allergens [5]. The concomitant reduction in the cellular expression and plasma concentrations of LIGHT is, therefore, of great interest. Consistent with the suppression of LIGHT and LTBR, there was a reduction in plasma TGF*β* which is responsible for the fibrosis and remodeling through epithelial-mesenchymal transformation [17]. There was also a significant suppression of plasma concentrations of MCP-1 (CCL-2). Our previously published data also demonstrate that plasma concentrations of RANTES (CCL-5) and eotaxin (CCL-11) and the expression of chemokine receptors CCR-2 and CCR-5 are also suppressed by an insulin infusion [12]. Chemokines attract monocytes and eosinophils and thus actively participate in the pathogenesis of allergic asthmatic inflammation. There was, however, no change in plasma TSLP concentrations.

Our data also show that insulin infusion suppresses the plasma concentration of NOM, suggestive of a reduction in NO generation, probably from iNOS. This observation is consistent with a previous demonstration that, in patients in intensive care, the infusion of insulin leads to a reduction in plasma concentration of NOM with a concomitant suppression of iNOS expression in the liver [18]. We have recently observed that the rapid increase in NO metabolites in plasma following the injection of endotoxin in normal subjects [19], indicative of a marked stimulation of iNOS from macrophages in the reticuloendothelial system, is totally inhibited by insulin given at a dose similar to that used in the current study. Since NO in exhaled air is considered to be indicative of intrabronchial inflammation in asthma and since this NO is generated by iNOS in bronchial macrophages [20], the suppression of NOM by insulin in our study suggests that insulin may also possibly be able to suppress NO generation by iNOS in bronchial macrophages and NO in exhaled air. ROS generation by MNC was also reduced by insulin infusion consistent with our previous observations [11]. It has also been shown previously that hydrocortisone has an ROS suppressive effect [21]. Furthermore, our recent data show that an injection of 300 mg of hydrocortisone (=60 mg prednisone) suppresses the expression of IL-4, LTBR, ADAM-33, and MMP-9 and the plasma concentration of MCP-1. The magnitude of this effect is similar to that of insulin infusion described in this study (unpublished observations). On the other hand, we have recently observed several paradoxical proinflammatory effects of this dose of hydrocortisone including an increase in plasma MMP-9 and HMG-B1 concentrations in parallel with increases in plasma glucose and FFA concentrations [22], all of which are suppressed by insulin. These data suggest that a combination of insulin and corticosteroids may form a future potential therapy for asthma.

The potential clinical significance of our observations is readily apparent. The insulin resistant proinflammatory states of obesity and type 2 diabetes are characterized by an increase in IL-4, MMP-9, LIGHT, and CCR-2 expression in MNC and MMP-9 and NOM concentrations in plasma [1]. Since IL-4, LIGHT, LTBR, TGF*β*, MMP-9, and NO are key mediators involved in the pathogenesis of allergy and asthma, the increase in their expression may contribute to the increased vulnerability and risk of asthma in the obese. It is relevant that weight reduction following gastric bypass surgery leads to a reversal of these increases in parallel with the restoration of insulin sensitivity [1]. By the fact that insulin infusion suppresses the expression of IL-4, ADAM-33, LIGHT, and LTBR and the plasma concentrations of NOM and MMP-9, the efficacy of insulin in the treatment of asthma needs to be assessed. In addition, insulin has been shown to suppress the plasma concentrations of several other relevant chemokines like RANTES (CCL-5) and the expression of their receptors like CCR-5 [12]. Its action may be additive to that of corticosteroids and may potentially reduce the dose of corticosteroids that is used to treat exacerbations of asthma in hospitalized patients. It is also relevant that insulin suppresses TLR-2 and TLR-4 [13], which mediate inflammatory responses in response to Gram positive bacteria and LPS, a product of Gram negative bacteria, respectively. In addition, it also suppresses the expression of TLR-7 and TLR-9 which get activated by RNA and DNA containing viruses, respectively [13]. There is evidence that these pattern recognition receptors contribute to the pathogenesis of asthma [23].

The fact that insulin resistant states lead to an increase in the expression of asthma related genes in spite of accompanying hyperinsulinemia and the fact that a low dose insulin infusion leads to the suppression of these genes need to be explained. The pathogenesis of insulin resistance is now thought to be due to an increase in proinflammatory genes [24] and free fatty acid (FFA) concentrations [25]. FFAs have also been shown to be proinflammatory [26]. These proinflammatory genes and FFAs interfere with insulin signal transduction [24, 27]. The increase in obesity related increase in asthma related genes is probably the result of systemic inflammation which concomitantly induces insulin resistance and hyperinsulinemia. However, this increase in insulin is not sufficient to suppress the inflammatory response. Insulin concentrations need to be increased beyond these concentrations by 3 to 4 times to induce an anti-inflammatory effect, be it the suppression of NF*κ*B or other inflammatory factors like chemokines or asthma related genes. Thus, the plasma concentrations of insulin need to overwhelm the resistance posed by the interference in insulin signaling caused by inflammation and FFAs.

There is an important limitation to this study since our investigations did not include patients with asthma and thus the observations cannot be applied to asthmatics immediately. However, they demonstrate for the first time the ability of insulin to suppress several of these key mediators following a short period of infusion in a group of patients with an increased risk of asthma. Clearly, a study investigating the effect of intravenous infusion on respiratory function in asthmatic patients needs to be carried out and is currently being planned at our center.

In conclusion, in patients with obesity and type 2 diabetes, insulin infusion leads to a suppression of the expression of IL-4, ADAM-33, LIGHT, and LTBR in parallel with a reduction in plasma NOM, TGF*β*, MCP-1, and MMP-9 concentrations. The potential role of intravenous infusion of insulin in the treatment of asthmatic patients needs to be investigated.

## Figures and Tables

**Table 1:** Change in inflammatory and oxidative stress mediators in MNC and serum following 2 U/hr insulin/dextrose infusion (insulin), dextrose alone (dextrose), or saline alone (saline) in obese T2DM for 4 hours. Data are presented as mean ± SE. ^*^ *P* < 0.05 by one-way RMANOVA (compared to baseline as absolute value and percent change); ^#^ *P* < 0.05 by two-way RMANOVA compared to control groups (as percent change from baseline).

	Group	0 h	2 h	4 h	6 h
Glucose (mg/dL)	Saline	134 ± 16	118 ± 14	112 ± 12	107 ± 11^*^
	Dextrose	133 ± 14	135 ± 11	125 ± 11	110 ± 10^*^
	Insulin	122 ± 15	114 ± 12	111 ± 10	109 ± 12^*^

Insulin (*μ*U/mL)	Saline	24.1 ± 4	25.4 ± 6	21.8 ± 5	22.4 ± 6
	Dextrose	27.6 ± 5	25.4 ± 6	22.9 ± 6	23.5 ± 7
	Insulin	20.1 ± 3	50.5 ± 8^∗#^	43.8 ± 9^∗#^	24.8 ± 4

MNC ROS generation (%)	Saline	100	97 ± 8	102 ± 7	96 ± 9
	Dextrose	100	109 ± 7	112 ± 8	104 ± 7
	Insulin	100	95 ± 6	82 ± 5^∗#^	112 ± 9

MCP-1 (ng/mL)	Saline	275 ± 36	277 ± 38	269 ± 34	262 ± 34
	Dextrose	256 ± 38	262 ± 33	258 ± 31	269 ± 36
	Insulin	270 ± 40	233 ± 30^*^	225 ± 29^∗#^	282 ± 41

MMP-9 (ng/mL)	Saline	354 ± 58	342 ± 49	339 ± 55	341 ± 47
	Dextrose	327 ± 63	312 ± 55	313 ± 57	315 ± 48
	Insulin	367 ± 58	338 ± 47	321 ± 31^∗#^	352 ± 44
